# Voltage Fluctuation in a Supercapacitor During a High-*g* Impact

**DOI:** 10.1038/srep38794

**Published:** 2016-12-13

**Authors:** Keren Dai, Xiaofeng Wang, Yajiang Yin, Chenglong Hao, Zheng You

**Affiliations:** 1 Collaborative Innovation Center for Micro/Nano Fabrication, Device and System, State Key Laboratory of Precision Measurement Technology and Instruments, Department of Precision Instrument, Tsinghua University, Beijing, 100084, China

## Abstract

Supercapacitors (SCs) are a type of energy storage device with high power density and long lifecycles. They have widespread applications, such as powering electric vehicles and micro scale devices. Working stability is one of the most important properties of SCs, and it is of significant importance to investigate the operational characteristics of SCs working under extreme conditions, particularly during high-*g* acceleration. In this paper, the failure mechanism of SCs upon high-*g* impact is thoroughly studied. Through an analysis of the intrinsic reaction mechanism during the high-*g* impact, a multi-faceted physics model is established. Additionally, a multi-field coupled kinetics simulation of the SC failure during a high-*g* impact is presented. Experimental tests are conducted that confirm the validity of the proposed model. The key factors of failure, such as discharge currents and discharging levels, are analyzed and discussed. Finally, a possible design is proposed to avoid the failure of SCs upon high-*g* impact.

With the increasing concern for the worldwide energy crisis and environmental pollution due to the depletion and burning of fossil fuels, there is an urgent demand for efficient, clean and sustainable energy sources. Supercapacitors or electrochemical capacitors represent a promising approach to meet the increasing power needs of both macro and microelectronic devices^1^,^2^. Compared with other energy storage methods, supercapacitors offer advantages such as high power density, good environmental adaptability and long cycle life^3^,^4^,^5^,^6^,^7^. To date, supercapacitors have been widely used as energy supplies for electric cars, subway trains and satellites. For these practical applications, supercapacitors may suffer many extreme working conditions, and their reliability is a serious concern that needs to be taken into consideration in application design and development. For example, the high-*g* acceleration (high acceleration up to tens of thousands times that of gravity) often occurs during the operation of electric cars, subway trains and satellites when they are speeding up, slowing down, turning, etc. As a result, the influences of high-*g* acceleration must be considered in order to insure normal and safe operation of these systems.

There have been reports about the reliability of supercapacitors and other electrochemical energy storage devices, such as lithium batteries, under high-*g* acceleration impacts. According to these studies, supercapacitors may suffer voltage fluctuations while discharging during a high-*g* impact^8^. However, the previous reports for supercapacitor failure under high-*g* impact are only based on experimental tests. Due to the lack of theoretical analysis, the kinetic mechanism of this failure phenomenon is still unclear. To improve the reliability of supercapacitors during high-*g* impacts, it is highly necessary to study the kinetic mechanism of this failure phenomenon.

In this paper, we focus on the electrical-chemical mechanism of the supercapacitor voltage fluctuation during a high-*g* impact process. For the first time, a multi-field coupled kinetics model is proposed to describe the failure mode during such a process. In this model, the electrolyte flow and ion redistribution caused by the high-*g* impact account for the voltage fluctuation, and the ion concentration field and electrical field during the impact process can be worked out completely by the model. Simulations of supercapacitor voltage and ion concentration response processes are realized, and experimental tests are conducted using a Machete Hammer, which verify the model. Based on the failure mechanism characterized by the model, an optimized design is proposed to improve supercapacitor reliability during high-*g* impact. This paper reveals the fundamental nature of the high-*g* impact failure of supercapacitors and has guiding significance for avoiding the supercapacitor failure in practical applications.

## High-*g* Response Model of Supercapacitors

**Figure 1:**
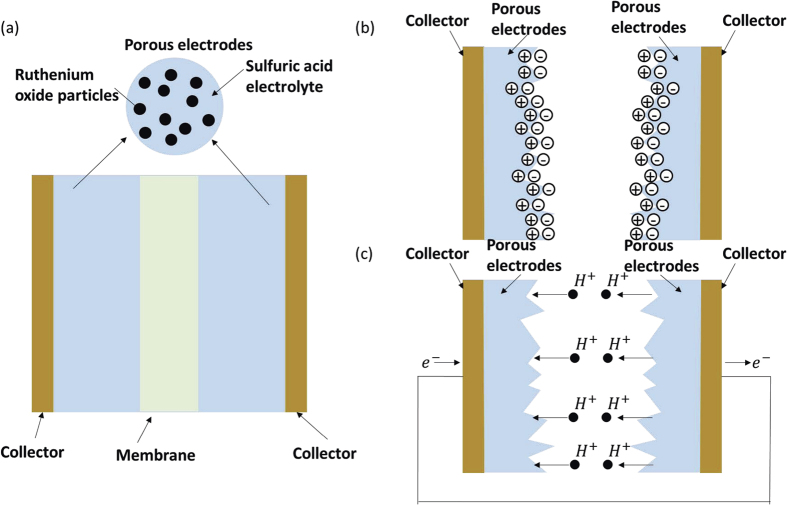
(**a**) Super capacitor structure diagram. (**b**) Electric double layer effect schematic. (**c**) Faraday process schematic.

A supercapacitor or electrochemical capacitor is composed of two electrodes, which are usually separated by an ion-permeable membrane, and an electrolyte ionically connecting both electrodes. It can be divided into two types: electrical double-layer capacitors and pseudo capacitors. The regular double-layer capacitance results from the potential-dependence of the surface density of charges stored electrostatically at the interfaces of the two electrodes. The pseudocapacitance results from Faradaic electron charge transfer with redox reactions, intercalation or electrosorption related to the electroactive materials. For supercapacitors in practical applications, the double-layer capacitors usually exhibit perhaps 1–5% of their capacitance as pseudocapacitance owing to the Faradaic reactivity of surface oxygen-functionalities. In contrast, pseudocapacitors usually exhibit 5–10% of their capacitance as electrostatic double-layer capacitance due to the ion adsorption/desorption in the interfacial areas. With this in mind, we established a dynamic model based on a typical supercapacitor, which is schematically shown in Fig. 1(a). The porous electrodes are usually made of materials with large surface areas, such as ruthenium oxide. A liquid electrolyte fully fills the pores of the electrodes and membrane. The energy storage mechanism of the supercapacitor is based on a combination of the electrical double layer effect and the Faraday process. The electric double layer effect stores energy at the surface of the electrodes. As shown in Fig. 1(b), the electrical double layer structure results from the electrical attraction between positive ions and negative ions. The Faraday process is a highly reversible redox reaction of the electroactive species, and energy storage can be realized through the resulting electron transfer, as shown in Fig. 1(c).

### Dynamic model for energy storage in a supercapacitor

The energy storage mechanism of a typical supercapacitor is shown in Fig. 1(b) and (c). Modeling and simulations are useful for theoretical research and optimizing designs of energy storage devices^9^. For example, a dynamic modeling simulation is the most accurate and effective way to study the mechanism and characteristics of the charging and discharging processes^10^,^11^,^12^,^13^,^14^,^15^,^16^,^17^, and it establishes a multi-faceted physics model composed of an electric field and an ion concentration field for the electrodes and the membrane, respectively.

First, in the porous electrode, the total current from the electrode phase to the electrolyte phase can be decomposed into the electric double layer current and the Faraday process current,


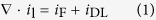


where *i*_l_ represents the surface current density of the electrolyte phase, and *i*_F_ and *i*_DL_ represent the current density that result from the Faraday process and the electric double layer effect, respectively.

For the electrode phase, the relation between the current and the potential is given by Ohm’s law,


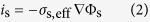


where *i*_s_, *σ*_s,eff_ and Φ_s_ represent the surface current density, the effective conductivity and the potential of the electrode phase. For the porous materials, all effective parameters are derived from their material parameters using the Bruggeman method. For example, 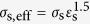, where *σ*_s_ represents the electrical conductivity of ruthenium oxide and *ε*_s_ represents the volume ratio of solid to total volume.

However, for the electrolyte phase of the electrode, Ohm’s law needs to be modified in light of the concentrated solution effect,


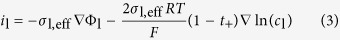


where *i*_l_, *σ*_l,eff_ and Φ_l_ represent the surface current density, the effective conductivity, and the potential of the electrolyte phase of the electrode, respectively. *t*_+_ and *c*_l_ are the transference number and ion concentration in the solution. *F, R* and *T* represent the Faraday constant, Universal gas constant, and the temperature in Kelvin, respectively.

The potential equation of the electrolyte phase can be obtained by combining equations (1) and (3),


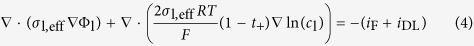


The relation ∇ · *i*_s_ + ∇ · *i*_l_ = 0 must hold due to charge conservation. The potential equation of the electrode phase can then be obtained by combining this conservation equation with equation (2),


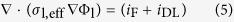


For the Faraday process, the binding and release of hydrogen ions at the surface of the ruthenium oxide is governed by classical porous electrode theory^13^. As shown in equation (6), the ion concentration field is influenced by the current and the transfer of ions,


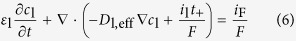


where *ε*_l_ and *D*_l,eff_ represent the porosity of the electrode and the hydrogen ions diffusion coefficient in the electrolyte, respectively.

In the membrane, *i*_s_ = 0 since it is an insulator. Thus, ∇ · *i*_l_ = 0. One can write the potential equation for the electrolyte phase in the membrane as,


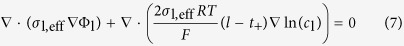


Similar to equation (6), the ion concentration equation for the electrolyte phase in the membrane can be obtained, since there is no Faraday current there.


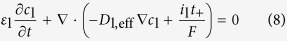


According to electrochemical theory, the Faraday current can be calculated as:


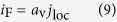


where *a*_v_ represents the specific surface area of the electrodes and *j*_loc_ represents the Faraday transfer current density. The *a*_v_ depends on porosity *ε*_l_, and the ruthenium oxide particle radius *r*_p_,


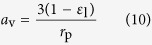


The *j*_loc_ term can be calculated by equation (10), according to the Butler-Volmer equation,


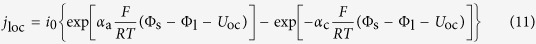


where *i*_0_ represents the Faraday exchange current density, *α*_a_ and *α*_c_ represent the transfer coefficients of the anode and cathode, and *U*_oc_ is the open-circuit potential of the Faraday process. The *U*_oc_ for the positive and negative electrodes are both dependent on the extent of the redox reactions,


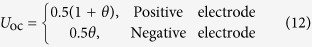


where *θ* represents the extent of the redox reactions, which can be represented by the ratio between the hydrogen ion concentration *c*_s_ of the electrode phase and the maximum achievable concentration *c*_s,max_,


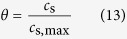


The *c*_s,max_ depends on the microstructure of ruthenium oxide,


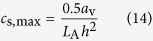


where *L*_A_ represents Avogadro’s number and *h* represents the lattice constant of ruthenium oxide.

According to the theory proposed by Kim and Popov^13^, we can obtain the conservation equations inside the ruthenium oxide particles (0 < *r* < *r*_p_),


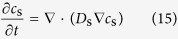


The boundary condition for equation (15) is,


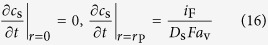


The current density *i*_DL_ created by the electric double layer effect is,


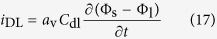


where *C*_dl_ represents the capacitance associated with the electric double layer effect.

Equations (1),(2),(3),(4),(5),(6),(7), (8),(9),(10),(11),(12),(13),(14),(15),(16),(17) fully describe the mechanism of the supercapacitor’s charging and discharging processes, and constitute a dynamic model for energy storage in the supercapacitor. A multi-faceted physics simulation for these processes can be realized by solving the model equations.

### Dynamic model for supercapacitor response based on the fluid and concentration fields

**Figure 2:**
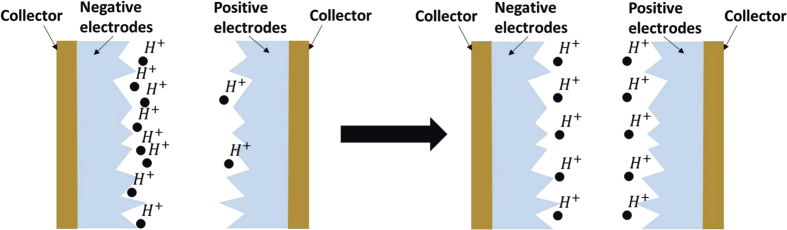
The impact response schematic of a supercapacitor.

To characterize the impact response of supercapacitors, the dynamic model for energy storage needs to be modified to account for the effects of a high-*g* process. In fact, an inhomogeneity in the hydrogen ion concentration always exists during the charging and discharging of the supercapacitor^18^, as shown in the left part of Fig. 2. The electrolyte will flow under the influence of a high-*g* impact, and this flow causes the hydrogen ions to redistribute homogeneously in a very short time, as shown in the right part of Fig. 2. The ion distribution caused by a high-*g* impact process has a significant influence on the performance of supercapacitors, which is discussed and modeled in the following section.

The inhomogeneity in the hydrogen ion concentration can be derived from a dynamic model proposed above. From equation (6) in the energy storage model of the supercapacitor, we can now consider the initial state before the supercapacitor begins charging or discharging. At the beginning, there is no Faraday current in the supercapacitor, and the hydrogen ion concentration in the electrolyte is homogeneous due to steady-state diffusion,


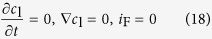


As the supercapacitor begins to charge or discharge, there are opposite Faraday currents in the positive electrode and negative electrodes, and hydrogen ions become bound to or released from these two electrodes.

For the charging process,


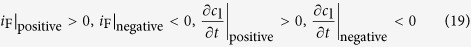


While for the discharging process,


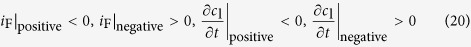


It can be concluded that the hydrogen ion concentrations of the two electrodes change in an opposing manner. As a result, the homogeneity of the ion concentration no longer holds once the charging or discharging process begins,


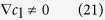


Fluid mechanics dictates that the liquid will flow due to the effects of gravity or acceleration^19^, and electrochemistry predicts that the electric field of the system may be affected by the fluid nature of the electrolyte^20^,^21^,^22^. The fluid field is controlled by the Navier-Stokes equations,


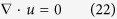



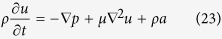


where *u, ρ, p, μ* and *a* represent the flow velocity, the density of the liquid, the pressure of the liquid, the dynamic viscosity, and the acceleration, respectively.

The ion concentration in the electrolyte not only is influenced by the Faraday current and the diffusion effect but also changes based on electrolyte flow. During an impact process, for example, a constant acceleration *a* lasts for a period of time *t*_g_. Equation (6) cannot accurately describe the change of ion concentration, and must be modified to include the fluid flow occurring during that time.

For the electrodes, equation (6) should be modified as:


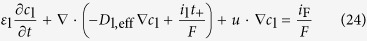


For the membrane,


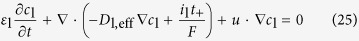


After replacing equations (6) and (8) with equations (24) and (25), a new model can be constructed with the electric field, ion concentration field, fluid field, and acceleration field. According to this model, the primary mechanism of the impact response is the ion redistribution process shown in Fig. 2. During the discharging process of the supercapacitor, the hydrogen ion concentration is larger at one electrode and smaller at the other due to ion release and binding during the Faraday process, as shown in the left part of Fig. 2. As the impact occurs, the electrolyte begins to flow and causes the ions to redistribute homogeneously, as shown in the right part of Fig. 2. Thus, it can be concluded that acceleration can directly influence the fluid field of the electrolyte and further change the ion concentration field and electric field of the supercapacitor, revealing the microscopic dynamic mechanism of the voltage fluctuations during high-*g* impact. The details of the response are discussed in the following section using simulation results.

**Figure 3:**
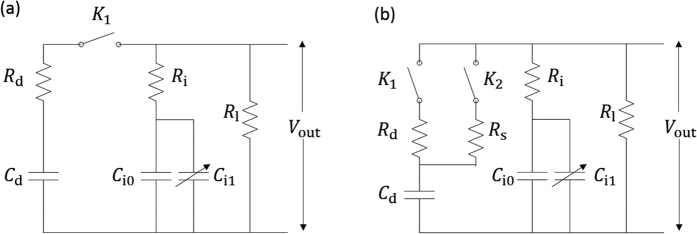
(**a**) Typical equivalent circuit model for the energy storage of a supercapacitor. (**b**) Equivalent circuit model for the impact response of a supercapacitor.

An equivalent circuit diagram is also proposed to help understand the impact response characteristics of supercapacitors intuitively. For a typical qualitative and semi-quantitative analysis of the charging and discharging process of a supercapacitor, the equivalent circuit model in Fig. 3(a) is commonly used^23^,^24^,^25^.

According to the equivalent circuit model shown in Fig. 3(a), the equivalent series capacitance *C*_i0_, *C*_i1_ and internal resistance *R*_i_ describe the fast transient response of the charging and discharging processes. In addition, the hysteresis loop composed of *C*_d_ and *R*_d_ characterizes the relaxation process of the supercapacitor caused by the ion redistribution effect. For practical supercapacitors, the relaxation process is relatively slow and can be ignored during charging and discharging, which means that the switch *k*_1_ between the hysteresis circuit and the main circuit will not close until the charging or discharging process is complete.

According to the mechanism discussed in equations (24) and (25), the impact process influences the supercapacitor through ion redistribution. The self-discharging theory of supercapacitors states that the relaxation process is also caused by ion redistribution. Therefore, in order to take the supercapacitor’s response to acceleration into account, we should modify the typical equivalent circuit model, similar to modeling the relaxation process, by adding another hysteresis circuit, as shown in Fig. 3(b). The impact response is modeled by the hysteresis circuit composed of *C*_d_ and *R*_s_. This new hysteresis circuit has a small response time, which means that 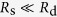 and that the relaxation time constant *R*_s_*C*_d_ is sufficiently small. When a high-*g* impact happens, the switch *k*_2_ closes, and this hysteresis circuit will be connected to the main circuit, resulting in a fast hysteresis process. The redistribution of charges between capacitors *C*_d_ and *C*_i0_, *C*_i1_ will result in a hysteresis current in the main circuit and fluctuate the output voltage *V*_out_.

The simulations in this paper are based on the dynamic model proposed above because it analyzes the micro scale impact response mechanism of the supercapacitor and is more accurate than the equivalent circuit model shown in Fig. 3(b).

## Simulation and Experiment Results

**Figure 4:**
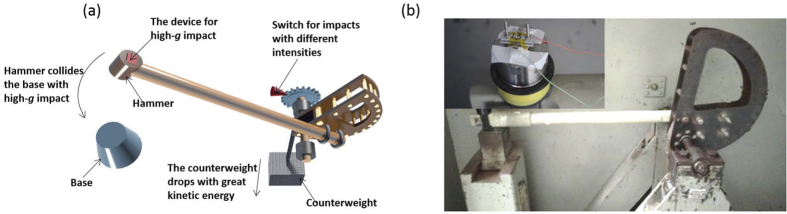
(**a**) The schematic of the Machete hammer. (**b**) The Machete Hammer and the supercapacitor for the high-*g* impacts tests.

Based on the dynamic model for the impact response of the supercapacitor that takes into consideration the fluid field and the concentration field, a multi-faceted physics dynamic simulation is implemented. The parameters in the simulations are shown in Table 1. Furthermore, to verify the validity of the dynamic model and the simulation, we tested the most important simulation conclusions experimentally by the Machete hammer test, which can provide accelerations of up to 30000 *g*. The Machete hammer test is commonly used in high-*g* impact experiments^26^ and can realize high-*g* impact by a violent collision process, with is caused by the huge kinetic energy of the counterweight, as shown in Fig. 4. We used a CHI 660 D electrochemical workstation to establish a supercapacitor high-*g* impact test platform. In the following part, we discuss the simulation and experimental results in detail.

**Figure 5:**
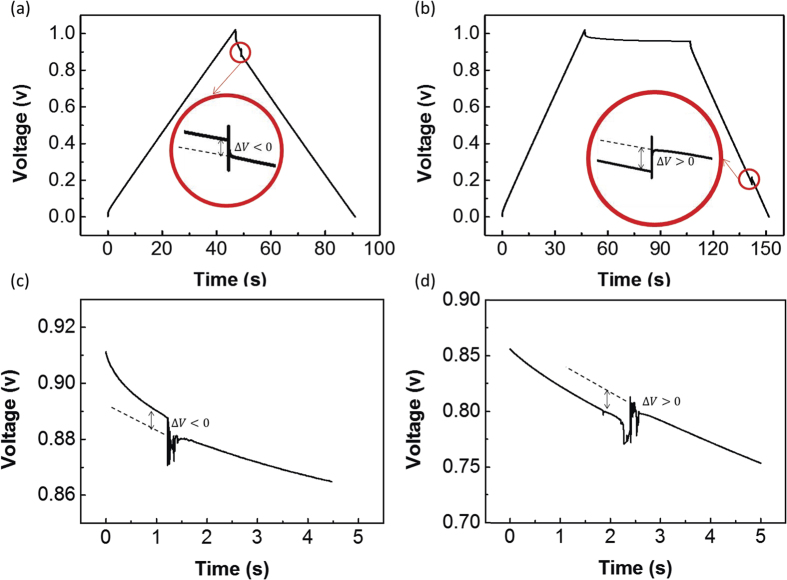
(**a**) The simulated voltage downward fluctuation for case 1. (**b**) The simulated voltage upward fluctuation for case 2. (**c**) The experiment measured voltage downward fluctuation for case 1. (**d**) The experiment measured voltage upward fluctuation for case 2.

Two typical impact response processes are simulated and their voltage responses are shown in Fig. 5(a) and (b). For case 1, the supercapacitor is charged with a large current, and the discharging process begins immediately after the supercapacitor is fully charged. Then, the supercapacitor suffers high-*g* impact. Figure 5(a) shows that the voltage of the supercapacitor fluctuates downward when the impact occurs under this condition; in other words, Δ*V* < 0, where Δ*V* represents the voltage increase during the impact process. For case 2, the supercapacitor begins to discharge after a long self-discharging process, and the high-*g* impact occurs during the discharging process. Figure 5(b) shows that the voltage of the supercapacitor fluctuates upward (Δ*V* > 0) when impact occurs in this condition, in contrast to case 1 in Fig. 5(a).

The experimental results also verify the two opposite voltage fluctuations for case 1 and case 2. Corresponding to case 1 in the simulation, the supercapacitor is first charged with a 20 mA current. The discharging process begins immediately after the supercapacitor is fully charged, and the high-*g* impact occurs shortly after the discharge begins. The experimental results clearly show the voltage drop (Δ*V* < 0) in Fig. 5(c). Corresponding to case 2 of the simulation, the supercapacitor is maintained at a constant voltage for a long time after it is fully charged. After the long period of constant voltage, the supercapacitor begins to discharge with a 20 mA current. The experimental results clearly show the voltage increase (Δ*V* > 0) in Fig. 5(d). The experimental phenomena shown in Fig. 5(c) and (d) are highly consistent with the simulation phenomenon shown in Fig. 5(a) and (b) and verify the validity of the proposed model.

To understand the nature of the two different failure phenomenon shown in Fig. 5, it is necessary to further study the changes of hydrogen ion concentration distribution in the electrolyte during the impact process. It is difficult to measure the ion concentration distribution experimentally during the impact process because the distribution changes dramatically during a short process and the ion concentration measurements are challenging to implement under such extreme conditions. Thus, we calculated the ion concentration distribution by simulation based on the proposed model.

**Figure 6:**
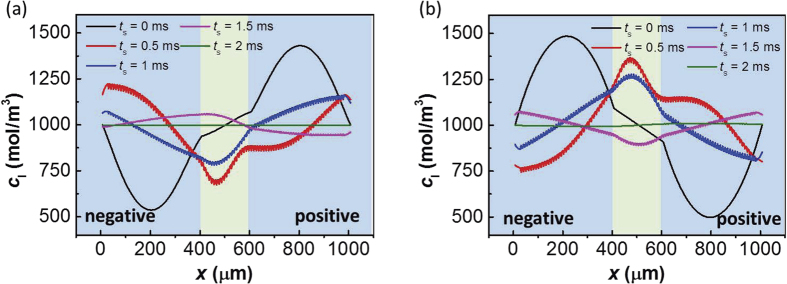
(**a**) Ion concentration distribution response simulation during an impact process for case 1. (**b**) Ion concentration distribution response simulation during an impact process for case 2. The *t*_*s*_ in this figure represents the time since the beginning of the impact process.

For case 1, while charging with a large current, the Faraday reaction releases hydrogen ions much faster than the rate of ion diffusion in the electrolyte. As a result, a large number of hydrogen ions are released and accumulated on the positive electrode, while hydrogen ions on the negative electrode are consumed at the same time. Before the high-*g* impact occurs, the hydrogen ion concentration at the positive electrode is much greater than at the negative electrode, as shown by the black line in Fig. 6(a). After the impact begins, the electrolyte flow quickly changes the distribution of the hydrogen ions in the electrolyte, and the ion concentration returns to a homogeneous state in a short time. This process is shown in Fig. 6(a), where *t*_s_ represents the time from the beginning of the impact. According to equation (5), the ion concentration *c*_l_ directly influences the electrolyte potential Φ_l_, which impacts the output voltage of supercapacitor. This voltage fluctuation can be attributed to the electrolyte ions redistribution effect^18^. During the discharging process of the supercapacitor, hydrogen ions are bound to the positive electrode. The impact process makes the hydrogen ion concentration more homogeneous and reduces the hydrogen ion concentration at the positive electrode. The decrease of hydrogen ions at the positive electrode reduces the discharging capacity of the supercapacitor and makes the output voltage drop.

In contrast, the ion concentration distribution of case 2, as shown in Fig. 6(b), has already become homogeneous after a long self-discharging process, according to the self-discharging theory of supercapacitors^18^. Then, as the supercapacitor discharges with a large current, the ion concentration at the negative electrode significantly exceeds that of the positive electrode, as shown by the black line in Fig. 6(b). During the impact process, the ion concentration becomes homogeneous in a short time. According to equation (5), the influence of the ion concentration *c*_l_ on the electric field depends on the value of ∇ln(*c*_l_). Due to the opposite values of ∇ln(*c*_l_) in Fig. 6(a) and (b), the voltage fluctuations in case 2 are opposite to that in case 1. As shown in Fig. 5(b), there is an upward voltage fluctuation, rather than a downward one, during the impact process. Based on the electrolyte ion redistribution, the electrolyte flow resulting from a high-*g* impact makes the hydrogen ion concentration more homogeneous and increases the hydrogen ion concentration at the positive electrode. As the electrolyte provides more hydrogen ions for the Faraday process, the discharge capacity is improved, and the output voltage increases.

To summarize, the voltage fluctuation is determined by the ion concentration distribution at the time of the impact. If the hydrogen ions are concentrated at the positive electrode at the initial time (*c*_l_|_positive_ > *c*_l_|_negative_), the voltage fluctuates downward (Δ*V* < 0) during the impact process, as shown in the Figs 5(a) and 6(a). Conversely, as shown in Figs 5(b) and 6(b), when the hydrogen ions are concentrated at the negative electrode at the initial time (*c*_l_|_positive_ < *c*_l_|_negative_), the voltage fluctuates upward (Δ*V* > 0) during the impact process.

**Figure 7:**
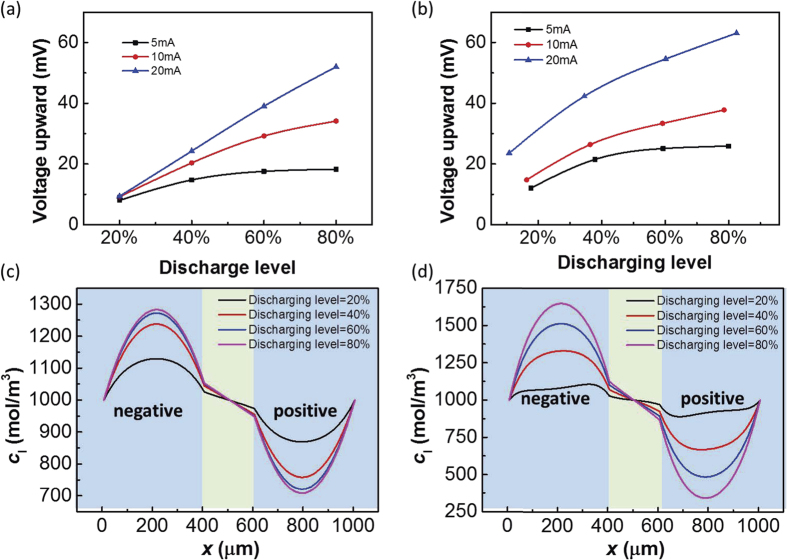
(**a**) The simulated amplitudes of voltage fluctuations with different discharge currents and different discharging levels. (**b**) The experimentally measured amplitudes of voltage fluctuations with different discharge currents and different discharging levels. (**c**) Ion concentration simulation for a discharge current of 5 mA. (**d**) Ion concentration simulation for a discharge current of 20 mA.

The analysis above demonstrates that the voltage fluctuation generated by the impact process depends on the hydrogen ion concentration of the electrolyte. Further simulation results will elaborate on this conclusion in detail. Considering the supercapacitors used in practical applications, most of them will not begin to discharge until they are fully self-discharged. For this reason, the following simulation will focus on the case 2 discussed above. Figure 7(a) shows the voltage fluctuations with different discharging currents and different discharging levels when the high-*g* impacts occur. We define the discharging level as the ratio of the output voltage when the high-*g* impact occurs to the maximum voltage when the supercapacitor is fully charged. The results in Fig. 7(a) show the character of the impact response. First, for the same discharging current, as the discharging level increases, the voltage fluctuation becomes larger. Second, for a larger discharging current, the voltage increase is larger. Third, for relatively small discharging currents, when the discharging level is low, the voltage increase is approximately a linear function of the discharging level. However, as the discharging level continues to increase and approaches 1, the amplitude of voltage fluctuation increases relatively slowly and finally approaches a constant value.

Corresponding to the simulation results in Fig. 7(a), the amplitudes of voltage fluctuation under different discharge currents and discharging levels are experimentally measured. The experimental results in Fig. 7(b) show that the amplitude of voltage fluctuation increases as the discharging level and discharging current increase. According to the experimental results in Fig. 7(b), when the discharging current is small, as the discharging level increases, the amplitude of the voltage fluctuation increases slower and slower, similar to the convergence phenomenon illustrated by the black line of Fig. 7(a). The only difference between the simulation and experimental results is that the experimentally measured voltage fluctuation amplitudes are always larger than the simulated ones under certain discharging current and discharging levels. This phenomenon is due to the piezoresistive effect of the supercapacitor electrodes. The porous electrode is piezoresistive and has less resistance during the high-*g* impact due to the huge pressure. The decrease of the electrode resistance reduces the voltage of the electrode. As a result, the output voltage of the supercapacitor will increase. This output voltage increase caused by the piezoresistive effect of the electrode accounts for the difference between the simulated and experimentally measured voltage fluctuation amplitudes. The qualitative consistency between the simulation (Fig. 7(a)) and experiment (Fig. 7(b)) further proves the validity of the proposed model.

These important findings from simulations and experiments can be explained by the ion concentration simulations in Fig. 7(c) and (d). First, the concentration difference results from the accumulation of Faraday current. Thus, as the discharging level increases, more ions accumulate at the negative electrode, and the concentration difference becomes larger. As a result, homogenization of the concentration field caused by the high-*g* impact process will influence the electric field more significantly. Second, a concentration difference arises from the fact that the ion release rate in the Faraday reaction exceeds the diffusion rate. As the discharging current becomes larger, the Faraday reaction becomes more dramatic, and the concentration difference becomes larger. Third, according to equation (18), the rate of diffusion depends on the concentration difference. As the concentration difference becomes sufficiently large, the diffusion effect can offset the ion release in the Faraday reaction and keep the concentration stable, as shown in Fig. 7(c). Thus, the voltage fluctuation approaches a constant value, although the discharging level continues to increase.

The simulation and experimental results both show that supercapacitors suffer voltage fluctuations during high-*g* impact processes. The inhomogeneity of the ion concentration and the electrolyte flow are the two primary reasons for these voltage fluctuations. To avoid this failure during high-*g* impact, it is necessary to reduce the inhomogeneity of ion concentration or the electrolyte flow, which can be achieved by using a large diffusion-coefficient electrolyte and a solid electrolyte, respectively. To reduce the inhomogeneity of the ion concentration, it is worthwhile to use an electrolyte with a large diffusion coefficient. According to equations (24) and (25), such an electrolyte will allow ions to diffuse more quickly and reduce the ion inhomogeneity, resulting in a smaller effect by a high-*g* impact. It is also helpful to use a solid electrolyte, which does not flow when the impact occurs, unlike a liquid one, so that the ion concentration and output voltage can remain stable during a short duration impact process.

## Conclusions

This paper investigates the failure of a supercapacitor during a high-*g* impact. The impact response mechanism of the supercapacitor is analyzed, and a dynamic model for the voltage response is established. Based on this model, a multi-faceted physics simulation of the supercapacitor under a high-*g* impact is achieved, and the experimental results verify the proposed model and simulation. According to this model, the high-*g* impact causes the electrolyte to flow and results in a change of ion concentration distribution, which further influences the electric field and makes the supercapacitor’s voltage fluctuate. Simulation results indicate that the inhomogeneity of ion concentration and the electrolyte flow are the two primary factors that control these voltage fluctuations. There will be larger fluctuations when there is a larger inhomogeneity of the ion concentration. To avoid this failure during high-*g* impacts, it is suggested that electrolytes with a large diffusion coefficient or a solid electrolyte be used for supercapacitors.

## Additional Information

**How to cite this article**: Dai, K. *et al*. Voltage Fluctuation in a Supercapacitor During a High-*g* Impact. *Sci. Rep.* **6**, 38794; doi: 10.1038/srep38794 (2016).

**Publisher's note:** Springer Nature remains neutral with regard to jurisdictional claims in published maps and institutional affiliations.

## Figures and Tables

**Table 1:** Values of parameters for the simulation.

parameter	value	parameter	value
*i*_0_	10^−5^ A/cm^2^	*T*	298.15 K
*α*_a_ & *α*_c_	0.5	*D*_s_	2 × 10^−12^ m^2^/s
*ε*_l_ for electrode	0.25	*D*_l_	2 × 10^−12^ m^2^/s
*ε*_l_ for membrane	0.7	*L*_A_	6.02 × 10^23^/mol
*r*_p_	1.5 × 10^−6^ cm	*h*	4 × 10^−8^ cm
*C*_dl_	2 × 10^−5^ F/cm^2^	*t*+	0.4
*σ*_s_	10^5^ S/cm	*ρ*	1.07 × 10^3^ kg/m^3^
*σ*_l_	0.8 S/cm	*μ*	10^−2^ Pa · s
*F*	96485 C/mol	*a*	1000 *g*
*R*	8.314 J/mol · K	*t*_g_	100 ms
